# Are Morphometric and Biomechanical Characteristics of Lumbar Multifidus Related to Pain Intensity or Disability in People With Chronic Low Back Pain After Considering Psychological Factors or Insomnia?

**DOI:** 10.3389/fpsyt.2022.809891

**Published:** 2022-04-15

**Authors:** Sabina M. Pinto, Jason P. Y. Cheung, Dino Samartzis, Jaro Karppinen, Yong-ping Zheng, Marco Y. C. Pang, Arnold Y. L. Wong

**Affiliations:** ^1^Department of Rehabilitation Sciences, The Hong Kong Polytechnic University, Kowloon, Hong Kong SAR, China; ^2^Department of Orthopaedics and Traumatology, The University of Hong Kong, Pokfulam, Hong Kong SAR, China; ^3^Department of Orthopedic Surgery, Rush University Medical Center, Chicago, IL, United States; ^4^International Spine Research and Innovation Initiative, Rush University Medical Center, Chicago, IL, United States; ^5^Medical Research Center Oulu, Oulu University Hospital and University of Oulu, Oulu, Finland; ^6^Rehabilitation Services of South Karelia Social and Health Care District, Lappeenranta, Finland; ^7^Finnish Institute of Occupational Health, Oulu, Finland; ^8^Department of Biomedical Engineering, The Hong Kong Polytechnic University, Kowloon, Hong Kong SAR, China

**Keywords:** chronic low back pain, fear-avoidance beliefs, sleep disturbance, lumbar multifidus muscle, CLBP

## Abstract

**Introduction:**

Lumbar multifidus muscle (LMM) dysfunction is thought to be related to pain and/or disability in people with chronic low back pain (CLBP). Although psychosocial factors play a major role in pain/disability, they are seldom considered as confounders in analyzing the association between LMM and CLBP.

**Objectives:**

This study aimed to determine: (1) differences in psychological factors, insomnia, and LMM characteristics between people with and without CLBP; (2) associations between psychological factors, insomnia, or LMM characteristics and low back pain (LBP) intensity or LBP-related disability in people with CLBP; and (3) whether LMM characteristics are related to LBP symptoms in people with CLBP after considering confounders.

**Methods:**

Seventy-eight volunteers with CLBP and 73 without CLBP provided sociodemographic information, filled the 11-point numeric pain rating scale and Roland-Morris disability questionnaire (RMDQ). They completed the Hospital Anxiety and Depression Scale (HADS), Pain Catastrophizing Scale (PCS), Fear Avoidance Belief Questionnaire (FAB), and Insomnia Severity Index Scale (ISI). Resting and contracted thickness of LMM at L4-S1 levels were measured from brightness-mode ultrasound images. Percent thickness changes of LMM at L4-S1 levels during contraction were calculated. Resting LMM stiffness at L4-S1 was measured by shear wave elastography. Associations among LMM, psychosocial or insomnia parameters and clinical outcomes were analyzed by univariate and multivariate analyses.

**Results:**

People with CLBP demonstrated significantly higher LBP-intensity, RMDQ, HADS, FAB, PCS, and ISI scores than asymptomatic controls (*p* < 0.05). The former also had significantly smaller percent thickness changes of LMM at L4/L5 during contraction. LBP-intensity was positively related to scores of PCS-total, PCS-helplessness, FAB-total, FAB-work, and ISI in people with CLBP (*p* < 0.05). RMDQ scores were positively associated with the scores of HADS-total, HADS-depression, PCS-total, FAB-total, FAB-physical activity, PCS-helplessness, and ISI in people with CLBP (*p* < 0.05). FAB-work and ISI scores together explained 24% of LBP-intensity. FAB-total scores alone explained 34% of variance of LBP-related disability in people with CLBP.

**Conclusion:**

More fear-avoidance belief or insomnia is related to greater LBP-intensity and/or LBP-related disability in people with CLBP. Although people with CLBP were thought to have aberrant LMM morphometry/function, no LMM characteristics were related to LBP-intensity or LBP-related disability after considering other confounders.

## Introduction

Low back pain (LBP) affects approximately 80% of adults at least once in their lifetime and is one of the leading causes of disability globally (1). LBP is defined as pain or discomfort between the twelfth ribs and buttocks (2). Although most LBP cases recover spontaneously, some people with LBP may experience chronic low back pain (CLBP) lasting for 3 months or more (3). The point prevalence of CLBP in the United States has been documented to be 13.1% (4). CLBP is one of the major causes of exorbitant treatment costs, and indirect costs due to sick leaves in the United States (5).

Morphometric and functional changes in lumbar multifidus muscle (LMM) may be related to CLBP (6–8). Since LMM is a spinal stabilizer that provides approximately two-thirds of spinal stability (9), aberrant changes in morphometry [e.g., muscle atrophy (10, 11) or fatty infiltration (12, 13)] or functional deficits of LMM (e.g., altered muscle activity and/stiffness) (14–16) may be related to the development or maintenance of CLBP. For instance, Danneels et al. reported low levels of surface electromyography activity in LMM among people with CLBP as compared to healthy individuals. Similarly, Masaki et al. (16) reported that the average LMM stiffness of people with CLBP was significantly higher than that of asymptomatic controls. Higher LBP intensity was significantly associated with higher LMM stiffness among people with CLBP (16). However, because prior research investigating the associations between LMM characteristics and CLBP clinical outcomes did not consider the influences of other confounders, it remains unclear whether their associations persist after taking confounders into account.

Multiple confounding factors are known to be related to CLBP. Compared to healthy individuals, people with CLBP are 2.3 to 3.2 folds more likely to have comorbidities (e.g., depression, anxiety and insomnia) (5). Previous research has suggested that various psychological factors [e.g., anxiety, depression, pain catastrophizing, fear-avoidance beliefs (FAB), etc.] are associated with pain intensity and/or disability in people with CLBP (17–22). In addition to mood disturbances, impaired sleep has been reported in people with CLBP (23, 24). Approximately 55% of people with CLBP experience insomnia (25), which is defined as sleep disturbance or difficulty in initiating sleep (26). People with CLBP also demonstrated significantly poorer sleep quality/quantity than asymptomatic individuals (23, 27).

Given the above, it is conceivable that correlations between various characteristics (e.g., resting and contracted LMM thickness, percent thickness changes during contraction, and resting muscle stiffness) of LMM and clinical outcomes in patients with CLBP may be modified after considering various psychological and/or sleep-related factors. A better understanding of these associations can improve the clinical management of these patients. Therefore, the current study aimed to: (1) compare the psychological factors, insomnia, and LMM characteristics between people with and without CLBP; (2) quantify the correlations between various psychological factors, sleep disturbance, or LMM characteristics and clinical outcomes (intensity of LBP and LBP-related disability) in people with CLBP; and (3) determine whether LMM characteristics are related LBP or LBP-related disability in people with CLBP after considering other confounders.

## Materials and Methods

### Participants and Study Design

This case-control study was conducted in a university laboratory. Individuals aged between 18 and 65 years were eligible for the study. Participants with CLBP (*n* = 78) were recruited from a public hospital, while asymptomatic participants (*n* = 73) were recruited from the university campus. People with CLBP were recruited if: (1) they experienced non-specific CLBP [defined as pain not attributable to a specific cause (28)] with or without leg pain that lasted for 3 months or more (3), that required medical consultation; and (2) their LBP intensity was at least 5 out of 10 on an 11-point numeric pain rating scale (NPRS). Age-matched asymptomatic controls should not experience an episode of LBP in the last 24 months. Exclusion criteria for all participants were: history of neurological disease, systemic inflammatory disease, previous spinal surgery, spinal fractures/tumors, metabolic disease, confirmed or suspected pregnancy, and indication for spine surgery.

### Data Collection Procedures

Following the provision of informed written consent as suggested by the Human Subjects Ethics Sub-committee of the university (HSEAR20151027007-01), participants were instructed to complete a battery of questionnaires related to their demographics, Pain Intensity, LBP-related disability, fear-avoidance beliefs, pain catastrophizing, anxiety, depression, and insomnia.

#### Demographic Questionnaire

The questionnaire asked questions related to the participant’s age, gender, body mass index, education level, work status, married status, and smoking and drinking habits.

#### Standardized Questionnaires

Pain: An 11-point numeric pain rating scale (NPRS) was used to quantify LBP intensity, with “0” representing “no pain at all” and “10” representing “the worst imaginable pain” (29). Participants were asked to choose a number best represented: (1) the current level of pain; as well as (2) the least and (3) worst levels of pain during the past 24 h. The pain level over the past 24 h was estimated using the average of three ratings (30). The pain intensity level was categorized as mild (1–5), moderate (6–8) and severe (9–10) (31). A cut-off score of >4 is considered as the minimal clinically important change in people with CLBP (32). The scale has shown excellent test-retest reliability [intraclass correlation coefficient (ICC) = 0.99] in assessing pain intensity among people with musculoskeletal pain (33).

Low back pain-related disability: participants’ functional disability was assessed by the Hong Kong-Chinese version of the 24-item Roland-Morris Disability Questionnaire (RMDQ) (34). It evaluates the impact of LBP on daily function, with scores ranging from 0 to 24 (0 means no disability; 24 means maximum disability). From the total score, the disability was classified into mild (0–8), moderate (9–16), and high (17–24) severity (34). A cut-off score of >4 indicates people with dysfunctional LBP (35). RMDQ has demonstrated excellent test-retest reliability (ICC = 0.94) in assessing LBP-related disability in people with non-specific CLBP (34).

Mood: The Chinese version of the Hospital Anxiety and Depression Scale (HADS) was used to assess anxiety and depression (36). It consists of two 7-item subscales measuring anxiety (HADS-A) and depression (HADS-D). Each of the 14 items is scored from 0 to 3 (37). Total scores of <7, 8–10, 11–14, and 15–21 in each subscale indicate non-cases, mild, moderate, and severe problems, respectively (38). A cut-off value of >8 is considered as clinically significant scores in each subscale of anxiety or depression (39). For the total score, >13 is considered as clinically significant scores for both anxiety and depression (39). This questionnaire has shown excellent internal consistency (Cronbach’s alpha = 0.84) in evaluating anxiety and depression among Chinese patients with cancer and their family caregivers (40).

Pain catastrophizing: The Chinese version pain catastrophizing scale (PCS) was used to assess pain catastrophizing (41). This 13-item questionnaire consists of 3 subscales: rumination, magnification, and helplessness (41). Total PCS scores of 30 or above signify clinically significant pain catastrophizing in people with chronic pain (42). It has demonstrated excellent internal consistency for the total PCS score (α = 0.9) (41).

Fear-avoidance beliefs: The level of pain-related fear was evaluated by the Hong Kong-Chinese version of the 16-item Fear-Avoidance Beliefs Questionnaire (FAB). It has demonstrated excellent internal consistency (α = 0.8), reliability, and validity in measuring fear-avoidance beliefs in people with CLBP (17, 43). Each item was graded on a 7-point Likert-type scale (0 means completely disagree; 6 means completely agree). It consists of 2 subscales: (1) beliefs about damage from physical activity (FAB-PA) [4 items (2,3,4,5); score range: 0 to 24]; and (2) beliefs about damage from work-related activities (FAB-W) [7 items (6,7,9,10,11,12,15); score range: 0 to 42]. The remaining five items are excluded from the calculation. The FAB-PA subscale is classified as low (0–14) and high fear levels (15–24). The FAB-W subscale is also classified as low (0–33) and high fear levels (34–42). The overall total score was calculated by adding the score of both subscales (17). The cut-off scores of >13 and >29 for FAB-PA and FAB-W, respectively, have been reported to be predictive of poor clinical outcome (disability) in people with LBP (44). For FAB-Total, cut-off scores of ł48 are considered to predict persistent disability in the future (45).

Insomnia: The severity of insomnia was assessed by the Chinese version of the 7-item Insomnia Severity Index (ISI). Each item is rated on a 5-point Likert scale (e.g., 0 = no insomnia; 4 = very severe insomnia) (46). The total scores were interpreted as no insomnia (0–7), sub-threshold insomnia (8–14), moderate insomnia (15–21), and severe insomnia (22–28) (47). A cut-off value of 10 is considered to be optimal to detect insomnia in the community (48). The ISI has demonstrated good test-retest reliability (α = 0.88) in people with chronic pain (49).

#### Lumbar Multifidus Muscle Assessments

**FIGURE 1:**
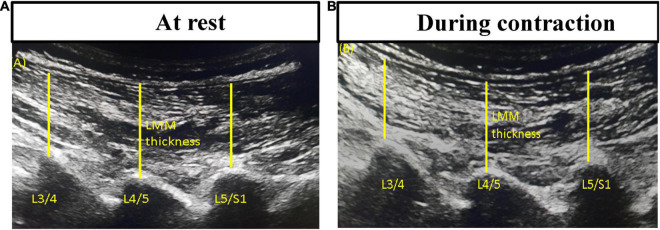
Thickness measurements of lumbar multifidus muscles using bright-mode ultrasound images **(A)** at rest and **(B)** during contraction.

Lumbar multifidus muscle morphometry and function: Bilateral parasagittal images of LMM at the L4/L5 and L5/S1 levels at rest and during submaximal contraction were captured with separate brightness-mode ultrasound videos on Supersonic Imagine^®^ (Aixplorer Innovative UltraFast™ Ultrasound Imaging, France). This non-invasive ultrasonography technique has been used to estimate muscle activation (50). It has shown good to excellent intra-examiner (ICC = 0.86–0.90) and inter-examiner (ICC = 0.86–0.93) reliability in evaluating resting/contracted thickness and percent thickness change in LMM (51, 52). The participant in the prone position performed contralateral leg lifts three times to touch a bar fixed at 5-cm height in order to elicit submaximal voluntary contraction of LMM (53). The lumbar curve at rest was maintained at around 10°. The resting and contracted LMM thicknesses in the recorded brightness-mode videos were then measured on the ultrasonography device. The thickness was determined from the distance between the posterior tip of the facet joint and the inside edge of the overlying fascia (Figure 1). The average of three measured thickness ratios (thickness contracted - thickness rest/thickness rest x 100%) of each LMM muscle was used for statistical analysis.

**FIGURE 2:**
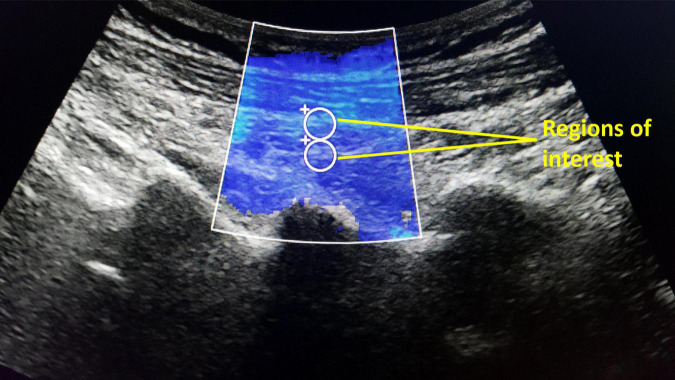
The supersonic shear wave imaging for lumbar multifidus stiffness measurements based on average pixel intensity within two regions of interest (5 mm diameter).

The shear modulus (stiffness) of bilateral LMM at the L4/L5 and L5/S1 levels of the participants were assessed at rest by supersonic shear wave imaging (SSI) function of Supersonic Imagine^®^. The resting LMM stiffness at each muscle level was measured thrice. A curved (1–6 MHz) SSI ultrasound probe was placed parallel to LMM fibers at the target level (54). The probe sent multiple ultrasound push beams focused on various depths to deform and to create shear waves in LMM. The machine detected the shear waves and generated the resulting 2-dimensional shear modulus color maps at 1 sample/second. On each map, two standardized circular regions of interest (ROIs) with 5 mm diameter were placed between 1 and 2 cm depth of the target LMM (Figure 2). The average pixel intensity within the ROIs on each map indicates the LMM shear modulus. The shear modulus (μ) within each ROI was automatically calculated by the software using the formula μ = ρv^2^, where ρ is the muscle mass density and v is shear wave speed (55). The resting LMM stiffness was estimated by averaging the shear modulus of each LMM muscle at rest.

### Data Analyses

Statistical analyses were performed using SPSS software (Version 26, IBM Corp., Armonk, NY, United States). Since Shapiro-Wilk tests indicated that our data was not normally distributed, non-parametric tests were used for data analyses. Descriptive statistics were conducted to summarize demographic characteristics (median and interquartile range) pain intensity, and RMDQ scores, HADS scores, FAB scores, PCS scores, ISI scores, while the mean and standard deviation were used to report LMM parameters in people with and without CLBP. Mann-Whitney U tests were used to compare between-group differences in psychological and insomnia scores. Linear mixed model analysis, which is robust for non-parametric data, was used for between-group comparisons of LMM characteristics after adjusting for age, gender and body mass index (BMI) (56). LMM characteristics. Spearman’s rank correlation coefficients were used to evaluate the relationships among demographic characteristics, pain intensity, RMDQ scores, HADS scores, FAB scores, PCS scores, ISI scores, and LMM stiffness and LMM thickness ratios. The strength of the correlation was classified as very weak (0.00–0.19), weak (0.20–0.39), moderate (0.40–0.59), strong (0.60–0.79), and very strong (0.80–1.0) (57). Partial correlation analyses between pain intensity and LMM parameters were performed by adjusting for psychological variables that were significantly related to pain intensity. Likewise, partial correlation analyses between LBP-related disability and LMM parameters were conducted by adjusting for psychological variables that significantly related to LBP-related disability. Psychological, insomnia, and LMM variables that demonstrated significant correlations with the 11-point NPRS or RMDQ score were then entered into two separate multiple linear regression models using a stepwise approach (*p* < 0.05 for entry, *p* > 0.10 for removal) to evaluate the relation between LMM characteristics and pain intensity or LBP-related disability in people with CLBP after accounting for various confounders. The significance level was set at *p* < 0.05 for all tests.

## Results

### Demographic Data

Demographic data of 78 participants with CLBP and 73 asymptomatic participants are shown in Table 1. There were no significant differences in age, body mass index, percentage of male, occupation, smoking status, and alcohol use, except for education levels and marital status between groups.

**TABLE 1:** Characteristics of participants with chronic low back pain (CLBP) and asymptomatic individuals [Median (interquartile range)].

Characteristics	CLBP	Asymptomatic
Age (years)	46.0 (35.8 to 54.0)	48.0 (30.0 to 54.5)
Body mass index (kg/m^2^)	23.0 (21.0 to 25.0)	22.0 (20.0 to 24.0)
Gender male *n* (%)	32 (41.0%)	36.6% (26)
Education level *n* (%)*		
Less than college	34 (44.7%)	20 (28.2%)
College or above	42 (55.3%)	51 (71.8%)
Occupation *n* (%)		
Employed	53 (74.7%)	50 (75.8%)
Unemployed/retired.	18 (25.4%)	16 (24.2%)
Marital status *n* (%)*		
Married	49 (66.2%)	30 (47.6%)
Others	25 (33.8%)	33 (52.4%)
Smoking status *n* (%)		
No	72 (94.7%)	69 (97.2%)
Yes	4 (5.3%)	2 (2.8%)
Alcohol use *n* (%)		
No	54 (71.1%)	53 (74.6%)
Yes	22 (28.9%)	18 (25.4%)

*Married and others (Unmarried/divorced/widowed).*

Calculation of p-values was performed using Mann-Whitney U test (for continuous variables) and chi-square test (for nominal and ordinal variables). *p < 0.05 for comparisons between people with CLBP and asymptomatic participants.

### Psychological and Sleep Parameters

People with CLBP demonstrated significantly higher pain intensity, disability, HADS, FAB, PCS, and ISI scores than asymptomatic participants (*p* < 0.05). Fifty percent and 61% of people with CLBP had clinically significant pain and disability, respectively, while 40 and 38% had clinically significant mood and fear-avoidance beliefs problems, respectively. Ten percent and 59% had clinically significant pain-catastrophizing and insomnia, respectively (Table 2).

**TABLE 2:** Summary of scores of psychological and sleep variables.

Variables	Measures	CLBP		Asymptomatic
		Scores [Median (IQR)]	Clinically significant n (%)	Scores [Median (IQR)]
Pain intensity	NPRS*	4.2 (3.0 to 5.6)	38 (50%)	0.0 (0.0 to 0.0)
Low back pain-related disability	RMDQ*	5.5 (3.0 to 9.0)	46 (60.5%)	0.0 (0.0 to 1.0)
Anxiety and depression	HADS Total*	11.5 (7.2 to 16.8)	30 (39.47%)	8.0 (4.0 to 12.0)
	HADS-A*	7.0 (4.0 to 8.0)	18 (23.68%)	4.0 (2.0 to 6.5)
	HADS-D*	5.0 (3.0 to 8.0)	18 (23.68%)	3.0 (1.0 to 6.0)
Fear-avoidance beliefs	FAB-Total*	44.0 (27.0 to 53.0)	29 (38.16%)	0.0 (0.0 to 22.0)
	FAB-PA*	18.0 (14.0 to 21.0)	59 (77.63%)	0.0 (0.0 to 11.3)
	FAB-Work*	22.0 (10.0 to 27.0)	14 (18.42%)	0.0 (0.0 to 8.0)
Pain-catastrophizing	PCS Total*	17.0 (8.0 to 26.0)	10 (13.2%)	2.0 (0.0 to 11.0)
	PCS-H*	7.0 (3.3 to 11.8)		1.0 (0.0 to 3.0)
	PCS-M*	4.0 (2.0 to 6.0)		1.0 (0.0 to 3.0)
	PCS-R*	6.0 (2.0 to 9.0)		0.0 (0.0 to 4.0)
Sleep	ISI*	12.0 (7.3 to 15.0)	45 (59.2%)	5.00 (3.0 to 11.00)

FAB, fear-avoidance belief questionnaire; FAB-PA, fear-avoidance beliefs-physical activity; FAB-W, fear-avoidance beliefs questionnaire-work; HADS, hospital anxiety and depression scale; HADS-A, hospital anxiety and depression scale- anxiety; HADS-D, hospital anxiety and depression scale-depression; IQR, interquartile range; ISI = insomnia severity scale; NPRS, numeric pain rating scale; PCS, pain catastrophizing. *p < 0.05 for comparison between people with CLBP and asymptomatic participants.

### Lumbar Multifidus Muscle Parameters

Between-group comparisons of LMM characteristics at L4/L5 and L5/S1 are reported in Table 3. After adjusting for age, gender and BMI, the percent thickness change of LMM during contraction at L4/L5 was significantly greater in asymptomatic participants than that in people with CLBP (*p* < 0.05). There were no significant differences in LMM resting thickness, contracted thickness, or LMM resting stiffness at both levels between people with and without CLBP. Likewise, the percent thickness change of LMM at L5/S1 during contraction was not statistically different between groups.

**TABLE 3:** Between-group comparisons of LMM parameters.

Variables	CLBP			Asymptomatic		
	Average	Right	Left	Average	Right	Left
LMM resting thickness at L4/L5 (cm)	2.63 ± 0.46	2.63 ± 0.49	2.63 ± 0.50	2.52 ± 0.43	2.49 ± 0.44	2.55 ± 0.49
LMM resting thickness at L5/S1 (cm)	2.74 ± 0.52	2.75 ± 0.55	2.74 ± 0.57	2.62 ± 0.46	2.61 ± 0.49	2.64 ± 0.48
LMM contracted thickness at L4/L5 (cm)	3.20 ± 0.51	3.20 ± 0.52	3.20 ± 0.54	3.16 ± 0.45	3.14 ± 0.47	3.17 ± 0.51
LMM contracted thickness at L5/S1 (cm)	3.11 ± 0.57	3.09 ± 0.58	3.13 ± 0.60	3.10 ± 0.44	3.12 ± 0.44	3.08 ± 0.47
Percent thickness change during contraction at L4/L5*	0.22 ± 0.81	0.22 ± 0.12	0.23 ± 0.11	0.27 ± 0.10	0.27 ± 0.12	0.26 ± 0.11
Percent thickness change during contraction at L5/S1	0.18 ± 0.11	0.17 ± 0.12	0.19 ± 0.15	0.18 ± 0.09	0.18 ± 0.10	0.17 ± 0.10
LMM resting stiffness at L4/L5 (kPa)	43.31 ± 21.53	43.71 ± 25.94	42.86 ± 26.75	41.27 ± 18.72	39.45 ± 20.22	43.09 ± 27.48
LMM resting stiffness at L5/S1 (kPa)	43.51 ± 21.16	42.40 ± 27.39	44.87 ± 24.74	41.91 ± 19.42	40.91 ± 25.31	42.90 ± 23.32

Adjusted for age, BMI, and gender, *p < 0.05 for comparison between people with CLBP and asymptomatic participants.

*CLBP, chronic low back pain; cm, centimeters; kPa, kilopascal; LMM, lumbar multifidus muscle.*

### Correlations Between Pain Intensity and Demographic, Psychological, or Lumbar Multifidus Muscle Parameters

None of the demographic variables were associated with pain intensity. Table 4 shows the interrelation among various psychological and sleep variables, LMM variables, LBP intensity, and LBP-related disability. Spearman’s correlation analyses showed that pain intensity was significantly but weakly correlated with PCS-Total scores (ρ = 0.29, *p* < 0.05), and was moderately correlated with the scores of PCS-H (ρ = 0.34, *p* < 0.05), FAB-Total (ρ = 0.30, *p* < 0.05), FAB-W (ρ = 0.39, *p* < 0.05), and ISI (ρ = 0.44, *p* < 0.05) in people with CLBP. Partial correlation analysis revealed no significant association between any LMM parameters and LBP intensity.

**TABLE 4:** The interrelations among various psychological and insomnia variables, lumbar multifidus muscle (LMM) variables, low back pain (LBP) intensity, and LBP-related disability in people with chronic LBP.

	Age	Gender	BMI	Education level	Occupation	Smoking	Alcohol use	Marital status	HADS-T	HADS-A	HADS-D	PCS Total	PCS-H	PCS-M	PCS-R	FAB- Total	FAB-PA	FAB-W	ISI	Thickness Rest L4/L5	Thickness Rest L5/S1	Contracted thickness L4/L5	Contracted thickness L5/S1	Percent thickness L4/L5	Percent thickness L5/S1	Stiffness at rest L4/L5	Stiffness at rest L5/S1
NPRS	0.20	−0.01	−0.19	−0.22	0.10	−0.02	0.07	0.06	0.21	0.15	0.21	0.29*	0.34*	0.23	0.20	0.30*	0.04	0.39*	0.44*	0.05	0.04	0.05	05	−0.10	−0.07	−0.16	−0.10
RMDQ	0.26*	0.02	0.13	−0.36*	−0.09	−0.05	−0.10	0.04	0.26*	0.20	0.28*	0.25*	0.33*	0.17	0.14	0.34*	0.24*	0.26*	0.24*	0.20	0.12	0.14	0.06	−0.21	0.00	−0.09	−0.12

Spearman rank correlation coefficient. *p < 0.05. FAB = fear-avoidance beliefs questionnaire; FAB-PA, fear-avoidance beliefs questionnaire-physical activity; FAB-W, fear-avoidance beliefs questionnaire work; HADS, hospital anxiety and depression scale; HADS-A, hospital anxiety and depression scale-anxiety; HADS-D, hospital anxiety and depression scale-depression; ISI = insomnia severity index; NPRS, numeric pain rating scale; PCS, pain catastrophizing scale; PCS-H, pain catastrophizing scale-helplessness; PCS-M, pain catastrophizing scale-magnification; PCS-R, pain catastrophizing scale-rumination; RMDQ, Roland Morris disability questionnaire.

*The Spearman correlation coefficient values can range from + 1 to −1 where + 1 indicates a perfect positive association of ranks, 0 indicates no association between ranks and −1 indicates perfect negative association of ranks. The strength of the correlation can be classified as very weak (0.00 to 0.19), weak (0.20 to 0.39), moderate (0.40 to 0.59), strong (0.60 to 0.79) and very strong (0.80 to 1.0).*

### Correlations Between Low Back Pain-Related Disability and Demographic, Psychological, or Lumbar Multifidus Muscle Parameters

Spearman’s correlation analyses showed that RMDQ scores were significantly, but weakly correlated with age (ρ = 0.26, *p* < 0.05), HADS-total (ρ = 0.26, *p* < 0.05), HADS-D (ρ = 0.28, *p* < 0.05), PCS-Total scores (ρ = 0.29, *p* < 0.05). RMDQ scores were also moderately correlated with the education level (ρ = −0.36, *p* < 0.05), FAB-Total scores (ρ = 0.34, *p* < 0.05), FAB-PA scores (ρ = 0.24, *p* < 0.05), FAB-W scores (ρ = 0.24, *p* < 0.05), PCS-H scores (ρ = 0.33, *p* < 0.05), ISI scores (ρ = 0.24, *p* < 0.05) in people with CLBP. No significant correlation was noted between RMDQ scores and any LMM parameters. Partial correlation analysis found that LMM parameters were not significantly related to LBP-related disability.

### Factors Explaining Low Back Pain-Intensity

Since no significant associations were noted between LMM parameters and LBP intensity or disability, only those psychological and sleep parameters were included in the regression models. Three independent variables were eligible for the entry to the regression model for predicting LBP intensity (FAB-W, PCS-H, and ISI scores). The final model accounted for approximately 24% of the variance of pain intensity (*R*^2^ = 0.241; adjusted *R*^2^ = 0.220). Specifically, high ISI scores and FAB-W scores were associated with higher pain intensity in people with CLBP (Table 5). The unique variance explained by each of the two independent variables indexed by the squared semi-partial correlations was relatively low (insomnia and fear-avoidance beliefs about work each only accounted for approximately 8% of the variance of pain intensity).

**TABLE 5:** Summary of stepwise regression model predicting numeric pain rating scale scores.

Model	B	SE-B	β
Constant	2.907	0.379	
ISI*	0.087	0.030	0.305
FAB-W*	0.040	0.014	0.301

*FAB-W, Fear-avoidance beliefs questionnaire-work subscale; ISI, Insomnia severity index.*

*B, regression coefficient; SE-B, standard error of B; β, standardized regression coefficient.*

The dependent variable was numeric pain rating scale scores. R^2^ = 0.241, Adjusted R^2^ = 0.220. F(2,73) = 11.60 *p < 0.001.

### Factors Explaining Low Back Pain-Related Disability

A two-stage hierarchical linear regression analysis was used to predict the level of disability reported by people with CLBP. In the first block, age and education levels were entered as a covariate; in the second block, HADS-D, FAB-T, PCS-H, and ISI scores were entered simultaneously as the primary variables of interest. Results of the hierarchical regression analysis are shown in Table 6. Only education level, entered on the first block, was a significant covariate, *F*(2, 73) = 4.035, *p* = 0.022. For the final block, the model was statistically significant *F*(6,69) = 5.926, *p* < 0.001, *R*^2^ = 0.340, Adjusted *R*^2^ = 0.283 and the FAB-T score accounted for 34% of the variance in RMDQ scores.

**TABLE 6:** Summary of hierarchical regression model predicting of Roland Morris Disability Questionnaire scores.

Block	*R* ^2^	Model	B	SE-B	β
1	0.100	Constant			
		Age	0.068	0.050	0.177
		Education (college or above)	−1.617	1.161	−0.183
2	0.340	Constant			
		FAB-Total*	0.063	0.032	0.241

*FAB-Total, fear-avoidance beliefs-Total.*

*B, regression coefficient; SE-B = standard error of B; β, standardized regression coefficient.*

The dependent variable was RMDQ scores. R^2^ = 0.340, Adjusted R^2^ = 0.283 *p ≤ 0.05.

## Discussion

Although individuals with CLBP had significantly smaller percent thickness change of LMM at the L4/L5 level during submaximal contraction than asymptomatic controls, no LMM parameters were significantly related to LBP-intensity or LBP-related disability in people with CLBP. Conversely, multiple psychological factors (e.g., pain catastrophizing and fear-avoidance beliefs) and insomnia were significantly related to LBP-intensity or LBP-related disability in individuals with CLBP. After considering various factors, FAB-W and ISI scores together explained 24% of the variance of pain intensity in individuals with CLBP. Similarly, FAB-Total scores explained 34% of the variance of LBP-related disability in people with CLBP. Taken together, our results lend support to the idea that the influence of psychological factors is more significant than the effect of LMM parameters on LBP intensity and LBP-related disability.

### Percent Thickness Change During Contraction

The average percent thickness change at L4/L5 during submaximal contraction in people with CLBP was less than that of asymptomatic participants accords with previous research by Kiesel et al. (15). They found significant differences in percent thickness change at L4/L5 between patients with CLBP and healthy individuals (15). However, our other LMM measurements showed no significant differences in resting or contracted LMM thickness at L4/L5 and L5/S1 levels, or no significant difference in resting LMM stiffness at L4/L5 and L5/S1 levels between people with and without CLBP. These findings concur with prior research. Sweeney et al. revealed no significant difference in resting thickness at L4/L5 and L5/S1 levels between patients with CLBP and healthy individuals (58). Wong et al. (51) demonstrated that the contracted thickness of LMM at L3/L4 and L4/L5 levels in patients with CLBP did not differ from that of asymptomatic individuals. Likewise, previous research found no significant difference in LMM stiffness at L4/L5 level between people with and without CLBP in different postures (59). Koppenhaver et al. also found that LMM resting stiffness at L4/L5 in patients with CLBP (*n* = 60) was comparable to that of healthy people (*n* = 60) (60). Although consistent non-significant findings may be attributed to the great variability in LMM thickness or stiffness among people with and without CLBP, it may also imply that certain pain related LMM changes only occur in some patient subgroups, or other LMM measurements (e.g., electromyography, functional cross-sectional area on magnetic resonance images) may be more sensitive to detect subtle differences in LMM parameters between people with and without CLBP.

Our non-significant correlations between the percent thickness change of LMM at the L4/L5 or L5/S1 level during contraction and pain or LBP-related disability (even after controlling for psychological factors) concurred with previous research (61). Zielinski et al. (61) reported no significant correlation between percent thickness change of LMM at L3/L4 and LBP or LBP-related disability in patients with CLBP at baseline. Interestingly, although their patients reported a significant reduction in disability after performing stabilization exercises, post-treatment improvements in Oswestry Low Back Pain Disability Questionnaire scores in these patients were not significantly related to the corresponding alteration in percent thickness change at the L3/L4 level. Similarly, two systematic reviews found that post-treatment changes in resting thickness, cross-sectional area or endurance of LMM were unrelated to the improvements in LBP or LBP-related disability in people with LBP (62, 63). Similar to our findings, a cross-sectional study found that neither LMM cross-sectional area nor thickness at the L4/L5 or L5/S1 level was significantly correlated to RMDQ scores among 45 people with CLBP (64). Another systematic review also found inconsistent evidence regarding the association between baseline percent thickness change of LMM during contraction and ensuing clinical outcomes after various non-surgical treatments (65). Given that the negative results may be ascribed to the suboptimal sensitivity of Brightness-mode ultrasonography in detecting selective impairments in the activation of deep LMM fibers among patients with CLBP (66), future studies should adopt other advanced technologies (e.g., intramuscular electromyography, ultrafast ultrasonography, multivoxel magnetic resonance spectroscopy, or diffusion magnetic resonance imaging) (67) to evaluate the potential relationship between LMM morphometry or function and clinical outcomes in patients with CLBP.

### Pain Catastrophizing

Similar to previous research, the current study found that pain catastrophizing was correlated with disability in people with CLBP (68, 69), but it did not predict LBP-related disability when it was concurrently evaluated together with other cognitive factors (70). Depression is one of the most common mental health conditions affecting people with chronic pain (71). Our study revealed that HAD-total scores and its depression subscale had weak positive correlations with LBP-related disability. These findings agreed with previous research. Hung et al. reported that the depression subscale was correlated with Oswestry Disability Index in people with CLBP (*n* = 225; *r* = 0.46) (72). Further, negative thoughts, low self-esteem, and decreased motivation for activity are symptoms of depression, which can negatively affect daily functioning and may contribute to disability (73).

### Fear-Avoidance Beliefs

Fear-avoidance beliefs are known to be related to pain intensity and LBP-related disability in patients with LBP (74–76). Mannion et al. (77) reported that reduced FAB total scores were significantly correlated with decreases in the disability scores. Numerous reasons may lead to the presence of fear-avoidance beliefs in patients. People experiencing pain may reduce their physical activity level because they fear that any movement may aggravate their pain intensity, which in turn becomes a vicious cycle leading to disability (78, 79). Fear may also disturb the neural control pathway for automaticity, resulting in deficits in trunk motor control and increased trunk variability during walking in uncontrolled daily-living environments (80), which may heighten the risk of LBP. Further, some people with CLBP believe that any painful movements may damage their spine or may intensify their suffering (81). Additionally, healthcare professionals’ fear-avoidance beliefs regarding LBP may inadvertently influence their patients’ beliefs (82). Therefore, healthcare professionals should evaluate and minimize their patients’ fear-avoidance behaviors. Given that psychological interventions (e.g., cognitive-behavior therapy) are significantly better than routine treatment (83), back-care advice (84) or exercises (85, 86) in reducing fear-avoidance beliefs in patients with LBP, healthcare professionals should be either trained to deliver behavioral psychological interventions (87) or refer indicated patients to psychologists for proper management.

### Insomnia

Almost 60% of our participants with CLBP reported clinically significant insomnia. Our findings also suggest that insomnia is one of the significant predictors of pain intensity in people with CLBP, which concurs with previous research that higher ISI scores were associated with higher pain intensity in people with CLBP (88). Similarly, a recent systematic review revealed low- to moderate-quality evidence that improved sleep quantity/quality is significantly related to improved LBP-related disability or reduced LBP in patients with CLBP (89). However, sleep disturbances and pain may affect each other reciprocally to form a vicious cycle because some brain regions (e.g., mesencephalic periaqueductal gray, thalamus, and raphe magnus) responsible for the initiation and maintenance of sleep are also involved in pain modulation (90).

Other factors may also explain the relation between sleep disturbances and pain. Different patients with chronic pain may have different circadian pain rhythms (91) and chronotypes (92). Some may have the highest pain intensity at wake-up that decreases during the day, while others may experience similarly high pain intensity in the morning that gradually decreases until it increases again from afternoon to night. Conversely, some may have the lowest pain intensity at waking and pain gradually increases over time (91). It has been postulated that those with high pain intensity in the morning may have suboptimal melatonin secretion at night, which may contribute to chronic sleep disturbances and increased pain perception in these patients (93). Interestingly, people with chronotype E (i.e., most active in the evening) experience a higher degree of musculoskeletal pain compared to those with chronotype M (i.e., most active in the morning). Collectively, circadian pain rhythms and chronotypes may have influence on pain (92).

In addition to the circadian pain rhythms, sleeplessness may affect pain sensitivity (94, 95). Insomnia is a known risk factor for developing back pain in asymptomatic individuals (96). Studies have found that sleep disturbance may affect the descending inhibitory pain pathways causing increased pain sensitivity (23, 97). Impaired sleep may also increase inflammatory cytokines that increase pain sensitivity (98, 99). A meta-analysis found that impaired sleep was significantly associated with higher levels of pro-inflammatory cytokines [e.g., interleukin (IL)-6] and biomarkers (e.g., C-reactive protein in the blood) (100) which might be related to more disability (101). Although the mechanisms underlying cytokines and disability remain to be determined, it is plausible that cytokines (e.g., IL-6, IL-1 and tumor necrosis factor-α) directly cause sarcopenia and functional impairments (102–105). Sleep-related changes in pain modulation may also limit functional abilities or activities of daily living in people with CLBP (106, 107). Regardless of the mechanisms, a large-scale prospective study involving 6,200 people with CLBP revealed that those with frequent sleeplessness at baseline had a lower probability of LBP recovery 11 years later (108). Therefore, preventing/reducing sleep-related problems in people with CLBP may improve their long-term prognosis. Future studies are warranted to evaluate the effects of sleep or pain interventions in modifying sleep, pain, and disability in people with CLBP.

### Limitations

There are several limitations in the current study. First, the cross-sectional study design cannot determine the causal relationship between various LMM, psychological, or sleep parameters and LBP or LBP-related disability in people with CLBP. Future longitudinal studies should determine whether the presence of one or more psychological factors are related to pain intensity or LBP-related disability at future follow-ups. Second, the duration of CLBP was not evaluated in the current study because many participants could not recall their durations of CLBP accurately, which could affect the relations between various factors and CLBP intensity and LBP-related disability. Third, data were collected from self-reported questionnaires, which may lead to social desirability bias and/or recall bias (109). That said, because all the self-reported questionnaires were validated screening tools for various psychological problems in patients with chronic pain (17, 40, 41, 49), they should be suitable for clinical practice and research. Fourth, since the current study only investigated the morphometric changes of LMM in patients with CLBP, the potential associations between aberrant changes in motor control or proprioception of LMM and pain among people CLBP (110, 111) remain uncertain. Future studies should evaluate the correlations between deficits in motor control, proprioception, and/or clinical spinal instability and LBP/LBP-related disability after controlling for psychological and sleep factors. Fifth, FAB, depression and anxiety has been reported to be positively correlated with neuroticism, which is one of the personality traits in people with CLBP (112). It was not within the context of the study to explore personality traits in people with CLBP. Future studies should investigate the influence of personality traits, psychological factors and LMM dysfunctions on LBP-related disability in people with CLBP.

### Strengths

This is the first study to evaluate the associations between various LMM parameters and clinical outcomes in patients with CLBP after adjusting for various psychological factors, insomnia, and demographic factors. Our findings highlight the necessity of assessing fear-avoidance beliefs and sleep disturbances in the routine clinical assessments of patients with CLBP, which may better manage these patients.

## Conclusion

Since aberrant LMM morphometry or stiffness may only occur in some, but not all, people with CLBP, the current study revealed no significant difference in LMM characteristics between people with and without CLBP (except greater percent thickness change of LMM at L4/L5 level during contraction in asymptomatic individuals). It may also explain why there were no significant associations between any LMM characteristics and LBP-intensity/LBP-related disability in people with CLBP. Conversely, fear-avoidance beliefs or insomnia closely related to pain intensity or disability in people with CLBP. As such, it is important for clinicians to use validated tools to screen for maladaptive fear and sleep disturbances in patients with CLBP so that timely treatments can be given.

## Data Availability Statement

The raw data supporting the conclusions of this article will be made available by the authors, without undue reservation.

## Ethics Statement

The studies involving human participants were reviewed and approved by Human Subjects Ethics Sub-committee of the University (HSEAR20151027007-01). The patients/participants provided their written informed consent to participate in this study.

## Author Contributions

SP and AW: conceptualization and design of the study, recruitment of participants, investigation, and data collection, and writing the original manuscript. AW: project administration. SP, AW, MP, JC, DS, and JK: analysis and interpretation of data. All authors critically revised the draft and approved the final version.

## Conflict of Interest

The authors declare that the research was conducted in the absence of any commercial or financial relationships that could be construed as a potential conflict of interest.

## Publisher’s Note

All claims expressed in this article are solely those of the authors and do not necessarily represent those of their affiliated organizations, or those of the publisher, the editors and the reviewers. Any product that may be evaluated in this article, or claim that may be made by its manufacturer, is not guaranteed or endorsed by the publisher.
