# Supplementary material for: Are Morphometric and Biomechanical Characteristics of Lumbar Multifidus Related to Pain Intensity or Disability in People With Chronic Low Back Pain After Considering Psychological Factors or Insomnia?
Source: Front Psychiatry. 2022 Apr 15;13:809891. doi: 10.3389/fpsyt.2022.809891 (PMC9053572; doi:10.3389/fpsyt.2022.809891)
Supplement: Supplementary file 1 [file Table_1.DOCX]

Supplementary Material

Supplementary Table

Table 1. Explanation of abbreviations used in this research paper

| Abbreviation Meaning |
| --- |
| LMM Lumbar multifidus muscle |
| CLBP Chronic low back pain |
| RMDQ Roland-Morris disability questionnaire |
| HADS Hospital Anxiety and Depression Scale |
| PCS Pain Catastrophizing Scale |
| FAB Fear Avoidance Belief Questionnaire |
| ISI Insomnia Severity Index Scale |
| LBP Low back pain |
| NPRS Numeric pain rating scale |
| CSA Cross-sectional area |
| IL Interleukin |
